# Could Hallucinogens Induce Permanent Pupillary Changes in (Ab)users? A Case Report from New Zealand

**DOI:** 10.1155/2017/2503762

**Published:** 2017-08-17

**Authors:** Ahmed Al-Imam

**Affiliations:** ^1^Novel Psychoactive Substances Unit, Doctoral College, Hertfordshire University, Hertfordshire, UK; ^2^College of Medicine, University of Baghdad, Baghdad, Iraq

## Abstract

An eighteen-year-old female patient of the Caucasian ethnicity from Australasia presented with a persistently dilated pupil causing her discomfort and occasional burning sensation when she is outdoors due to oversensitivity to sunlight. However, her pupillary reaction to light (pupillary light reflex) was intact. The patient is a known user of psychedelic substances (entheogens) including LSD, NBOMe, psilocybin, and DMT. The condition affects both eyes to the same extent. Thorough medical, neurological, and radiological examinations, including an EEG and an MRI of the head and neck region, were completely normal. All these tests failed to detect any pathophysiological or anatomical abnormalities. The patient is a known case of chronic endogenous depression in association with attention deficit hyperactivity disorder, for which she is taking citalopram and Ritalin, respectively. There was neither a family history nor a similar congenital condition in her family.

## 1. Introduction

Novel psychoactive substances (NPS), also known as research chemicals or designer drugs, are a group of substances, including chemicals that can either stimulate (stimulants) or inhibit (depressants) the nervous system, particularly the central nervous system [1, 2]. According to the classification scheme reported by the* European Monitoring Centre for Drugs and Drug Addiction *(EMCDDA), NPS can be categorised into cannabis and cannabimimetic, phenethylamines, cathinones, tryptamines, piperazine, and pipradrol derivatives and a 7th miscellaneous group which is composed predominantly of CNS stimulants [2, 3]. This taxonomy is based on the structural chemistry of 252 substances that were reported to the EMCDDA in between 1997 and 2012. The exponential rise in of the NPS phenomenon is considered to be correlated with the logarithmic growth of information and communication technology [4, 5].

The majority of these substances possess addictive properties. Hence, substance users and misusers may develop dependence syndrome, withdrawal manifestations, or adverse reactions [6]. These substances act on various neurotransmitters within the nervous system, including the central nervous system (CNS) and the peripheral nervous system (PNS) [2, 7]. The key neurotransmitters are monoamines including dopamine, serotonin, and catecholamine. In fact, NPS exert their effects via their highly selective affinity towards monoamine transporters (MATs); MATs include serotonin transporter (SERT), dopamine transporter (DAT), and norepinephrine (NET) [8, 9].

MATs are located just around the synaptic cleft (perisynaptically); they are responsible for the reuptake of monoamines back from the synaptic cleft into the cytoplasm of the presynaptic neurons [10, 11]. Therefore, the action of NPS on the body systems, both centrally and peripherally, can be attributed to changes achieved via MATs. NPS can induce several physiologic changes including ocular alterations [12], for example, morphometric variations in the dimension (diameter) of the pupillary aperture leading to either pupillary constriction (miosis) or dilation (mydriasis).

## 2. Case Report

**Figure 1:**
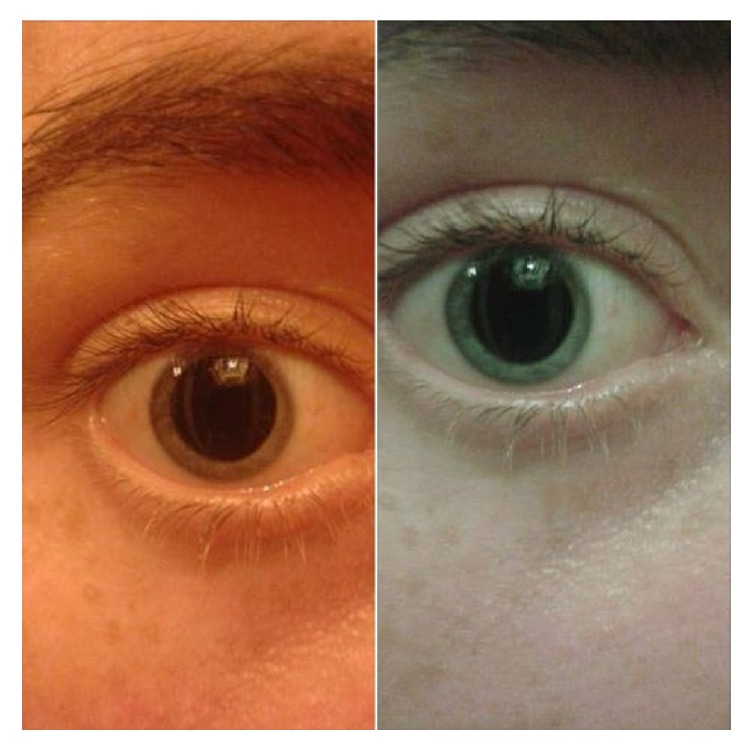
Dilated right pupil of a female psychedelics' user.

The patient is an 18-year-old female of the Caucasian ethnicity; she is from Australasia, specifically New Zealand; she has light coloured skin of Fitzpatrick type-1 category [13, 14]. She is a right-handed artist and of a potentially left-hemispheric cerebral dominance. She has a past history of substance use and misuse starting at the age of eleven years, smoking cannabis and hashish; at that age she developed an abnormally and continuously dilated pupil (Figure 1) leaving a thin rim of blue iridial tissue; the condition is affecting both eyes (bilaterally) though both pupils still react to light, including sunlight (i.e., pupillary light reflex is intact).

She had no complaints except for intolerance to sunlight, both direct and indirect, which mandates wearing* UV sunglasses* for optimal protection of the retina as advised by a specialist optometrist. Hence, the patient is almost unbothered by her condition; she described it by saying “*my eyes always look like this, I have constantly dilated pupils, haha, not even tripping*.” In fact, she considers her overall eye appearance as* sexier* than the normal eye. However, she was bothered of the potential eye and retinal damage from overexposure to sunlight.

The patient has correlated her eye condition with the use of hallucinogens, primarily with LSD (acid) and psilocybin mushrooms. Furthermore, her condition started at the age of eleven years in association with substance use. Hence, it is not congenital. In 1992, the 1st case of a bilateral congenital mydriasis was reported in the literature [15]. The patient has a past medical history, being treated with citalopram and Ritalin for the management of her chronic endogenous depression and attention deficit hyperactivity disorder (ADHD); she has been on these medications for several years. She also admitted using tramadol, opium, and opioid derivatives; these substances induce a paradoxical effect on her pupil leading to pupillary constriction. The patient had no prior history of head trauma, brain tumors, or any other neurological conditions. Furthermore, a thorough neurological and radiological exam was done; an MRI of the head and neck region was also entirely normal.

## 3. Discussion

In Renaissance Italy, Italian ladies used to apply a purified extract from the berries of* Atropa belladonna* as eye drops to both eyes; the purpose was to create artificially dilated pupils; it was considered a sign of beauty;* belladonna* is Italian for a* beautiful lady* [16, 17]. The extract contains anticholinergic substances including atropine, scopolamine, and hyoscyamine [16, 17]. In this case presentation, the pupillary changes (mydriasis) were brought by the pathophysiologic effect of possibly more than one substance, primarily hallucinogenic agents that own sympathomimetic or parasympathomimetic-related properties [18]. Though it may still be considered as a sign of beauty, it increases the sensitivity towards sunlight (photosensitivity) particularly in fair skin individuals and Caucasians of* Fitzpatrick skin type*-1 and type-2 [19].

Changes in the pupillary dimensions are brought up by either an increment or a decrement in its diameter via the action of iridial smooth muscles, both longitudinal (dilator pupillae) and circular (sphincter pupillae) [7]. The autonomic nervous system (ANS), an integral component of the PNS, is responsible for the automatic (visceral) control of the pupillary aperture [20]. The sympathetic nervous system (SNS) usually mediates pupillary dilation (mydriasis), while the parasympathetic nervous system, via the oculomotor nerve (cranial nerve III) and its modulatory effect over the ciliary autonomic ganglion, mediates pupillary constriction (miosis) [20, 21]. Changes in the sympathetic or parasympathetic tone (neuronal activity) are brought up by changes via reflex mechanism (as in pupillary light reflex), emotional changes affecting the limbic system and the diencephalon specifically the hypothalamus, and the modulation of the neuronal activity in the midbrain specifically pretectal region and the Edinger–Westphal nucleus (accessory oculomotor nucleus). The last nucleus houses the presynaptic (preganglionic) parasympathetic motor neurons of the oculomotor nerve which innervate the sphincter pupillae iridial muscles [7, 22]. Hence, this pathway, pretectal-accessory oculomotor nucleus, is a critical constituent of the pupillary light reflex; the afferent and efferent nerves of this pathway are the optic nerve and the oculomotor nerve, respectively; the reflex is considered to be a four-neuronal reflex pathway [23]. Accordingly, this reflex neuronal pathway controls the momentary changes* (pupillary unrest under ambient light)* in the pupillary aperture with a high accuracy and an ultimate speed (milliseconds) in response to variations in the illumination level of the surrounding environment [24, 25].

Certain pathophysiological changes can influence the pupillary aperture; these include the pupillary light reflex mechanism, the tone of SNS or PNS including their autonomic ganglion, and pathologies of the midbrain around the regions of the* cerebral aqueduct of Sylvius* including the regions of tectum and tegmentum, hypothalamic region, limbic system, and higher centres. For instance,* Horner's syndrome* is a condition in which damage affects the function of the upper segments (cervicothoracic) of the paravertebral sympathetic chain, leading to dilated pupil on the ipsilateral side in addition to ipsilateral eyelid ptosis and hemifacial anhidrosis [26]. Several conditions may result in Horner's syndrome, including central (CNS) and peripheral (PNS and ANS), including syringomyelia, multiple sclerosis, brain tumors, encephalitis, lateral medullary syndrome, cervical rib, thyroid tumors and thyroidectomy, bronchogenic carcinoma, tube thoracostomy, carotid artery dissection, cavernous sinus thrombosis, middle ear infections, sympathectomy, and nerve block procedures [26, 27]. All these pathological conditions operate either centrally at the level of the hypothalamospinal tract or at the presynaptic sympathetic neurons, or peripherally at the postsynaptic sympathetic neurons. Horner's syndrome can occur either unilaterally or bilaterally; several tests are used to diagnose this syndrome as in the* cocaine drop test* [28].

Several conditions and agents can cause mydriasis; these are injury to the eye and associated neural elements, anticholinergic medications and chemicals such as atropine and scopolamine, the elevated level of oxytocin hormone, and drug use and misuse [15, 29]. Drugs include cocaine (crack), MDMA (ecstasy), hallucinogens, methamphetamine (crystal meth), and Toradol (ketorolac). Hallucinogenic drugs and entheogen are not limited to LSD (acid), NBOMe (n-bomb), and dimethyltryptamine (DMT) [30]. Stimulants (as in cocaine) and hallucinogens act via increasing the levels of serotonin mainly by acting on SERT located centrally (CNS) [31]. In fact, these drugs that may lead to an overall increase in 5-hydroxytryptamine (serotonin) or a subsequent effect on 5-HT_2A_ receptor will exert a mydriatic effect, as in the case of psychedelics [11, 31]. Other conditions leading to abnormally dilated pupil include* benign episodic unilateral mydriasis*, cranial nerves neuropathy, traumatic brain injury, and mydriatic agents used for ophthalmologic examination such as tropicamide [32]. Oxytocin, the* love hormone*, can induce mild to moderate mydriatic effect; oxytocin is related to intimate emotional and social interactions. Hence, it increases in bursts during sexual intercourse; Pitocin (oxytocin) is also medicinally used to induce uterine contraction to either facilitate or induce a normal vaginal delivery [33, 34].

Similar cases were reported in drug fora, particularly from abusers of psychedelic substances including both males and females. One of the threads included this comment “*My friend has one pupil increased in size and one decreased. Permanently… It happened to him after I made a cacao from weed and we tripped our balls. It looks funny”* [35, 36]. In fact, some psychedelic users reported that they were able to control the size of their pupillary apertures voluntarily; a male has commented “*I can change the size of my pupils while looking in the mirror”* [35, 36]. This is remarkable given the fact that iridial muscles are strictly controlled involuntarily by autonomic innervation [36]. Perhaps, some neuronal (or neurochemical) modulation exists in psychedelic (ab)users. Contrary to psychedelic users, users and misusers of opium and opioid substances experience a constricted (miotic) pupil, or even a pinpoint pupillary aperture [37]. Heroin, fentanyl, codeine, methadone, and morphine act via stimulation of the PNS [38]. The New Zealander patient presented in this manuscript admitted taking these substances (opioids) too; she has also confirmed that her pupils could still react with some degree of constriction. She commented saying “*I do take tramadol and codeine as well, but they do the opposite to the eyes*.”

Clinical examination, including a thorough neurological exam, failed to detect any abnormalities apart from the bilaterally dilated pupils. Furthermore, MRI of the regions of the craniocervical and thoracic region could not detect any pathology. It is likely that changes may exist at a cellular level that cannot be detected with the conventional methods, or at the centrally located nuclei in the midbrain and the hypothalamus. Functional MRI (fMRI) can be useful to detect these changes, but it was not available in the medical institute at which the patient was examined [39, 40]. Transcranial magnetic stimulation (TMS) might be of value in detecting lesions in the limbic system, temporal lobe, or the prefrontal cortex. However, TMS is not suitable to detect deep-seated lesions in the hypothalamus or the midbrain [41, 42].

This level-of-evidence of this manuscript is level-5 in accordance with the classification system imposed by the* Oxford Centre for Evidence-Based Medicine* (CEBM) [43, 44].

## 4. Conclusion

The case of the New Zealander female presented in this manuscript is one of the few cases documented in the body of literature. The patient had a frequent use of NPS, psychedelics, and other psychoactive chemicals including antidepressant medications. It can be inferred that the burden of (ab)use of hallucinogenic and other NPS chemicals is not to be underrated, particularly in the developed world, including Australasia. These substances can be abused as early as childhood leading to irreversible consequences including adverse pathophysiological changes of body systems, dependence syndrome, incidents of intoxications, fatalities, and sudden death. The magnitude of these hazards is obscure in relation to the developing countries, including the Middle East, Asia, Africa, and Latin America. In connection with the Middle East, more in-depth epidemiological investigations are mandatory to infer an estimate in relation to the spread of psychedelic (ab)use.
